# A molecular inversion probe-based next-generation sequencing panel to detect germline mutations in Chinese early-onset colorectal cancer patients

**DOI:** 10.18632/oncotarget.15593

**Published:** 2017-02-21

**Authors:** Junxiao Zhang, Xiaoyan Wang, Richarda M de Voer, Jayne Y. Hehir-Kwa, Eveline J Kamping, Robbert D.A. Weren, Marcel Nelen, Alexander Hoischen, Marjolijn J.L. Ligtenberg, Nicoline Hoogerbrugge, Xiangling Yang, Zihuan Yang, Xinjuan Fan, Lei Wang, Huanliang Liu, Jianping Wang, Roland P. Kuiper, Ad Geurts van Kessel

**Affiliations:** ^1^ Department of Human Genetics, Radboud University Medical Center, Radboud Institute for Molecular Life Sciences, Nijmegen, The Netherlands; ^2^ Guangdong Provincial Key Laboratory of Colorectal and Pelvic Floor Diseases, Guangdong Institute of Gastroenterology and the Sixth Affiliated Hospital, Sun Yat-Sen University, Guangzhou, Guangdong, China; ^3^ Department of Pathology, Radboud University Medical Center, Nijmegen, The Netherlands; ^4^ Department of Pathology, the Sixth Affiliated Hospital, Sun Yat-Sen University, Guangzhou, Guangdong, China; ^5^ Department of Clinical Laboratory, the Sixth Affiliated Hospital, Sun Yat-Sen University, Guangzhou, Guangdong, China; ^6^ Princess Máxima Center for Pediatric Oncology, Utrecht, The Netherlands

**Keywords:** molecular inversion probes, early-onset colorectal cancer, Mendelian colorectal cancer predisposition syndromes, next-generation sequencing

## Abstract

The currently known Mendelian colorectal cancer (CRC) predisposition syndromes account for ∼5–10% of all CRC cases, and are caused by inherited germline mutations in single CRC predisposing genes. Using molecular inversion probes (MIPs), we designed a targeted next-generation sequencing panel to identify mutations in seven CRC predisposing genes: *APC, MLH1, MSH2, MSH6, PMS2, MUTYH* and *NTHL1*. From a consecutive series of 2,371 Chinese CRC patients, 140 familial and non-familial cases were selected that were diagnosed with CRC at or below the age of 35 years. Through MIP-based sequencing we identified pathogenic variants in six genes in 16 out of the 140 (11.4%) patients selected. In 10 patients, known pathogenic mutations in *APC* (five patients), *MLH1* (three patients), or *MSH2* (two patients) were identified. Three additional patients were found to carry novel, likely pathogenic truncating (*n* = 2) and missense (*n* = 1) mutations in the *MSH2* gene and a concomitant loss of expression of both the MSH2 and MSH6 proteins in their respective tumor tissues. From our data, we conclude that targeted MIP-based sequencing is a reliable and cost-efficient approach to identify patients with a Mendelian CRC syndrome.

## INTRODUCTION

Colorectal cancer (CRC; MIM 114500) is the third most common cancer in males and the second in females worldwide, with 1.2 million patients diagnosed annually [1]. In China, CRC is the fifth most commonly diagnosed cancer and the fifth leading cause of cancer-related death among both men and women, with an estimated 376,300 new patients and 191,000 deaths in 2015. Moreover, the age-standardized CRC incidence and mortality rates in China have shown a clear upward trend in recent years [2]. A family history of CRC or an early age at diagnosis are indications for a genetic predisposition. Genetic factors are estimated to account for the development of ∼30% of all CRCs [3]. The currently known Mendelian CRC predisposition syndromes, caused by germline mutations in single predisposing genes, account for ∼5–10% of all CRCs [4]. Examples of autosomal dominant Mendelian CRC syndromes are Lynch syndrome (LS) caused by mutations in the mismatch repair (MMR) genes *MLH1*, *MSH2*, *MSH6* or *PMS2*, familial adenomatous polyposis (FAP) caused by mutations in the *APC* gene and polymerase proofreading-associated polyposis (PPAP) caused by mutations in the exonuclease domain of the *POLE* or *POLD1* genes. Thus far, two autosomal recessive Mendelian CRC syndromes have been described, namely MUTYH-associated polyposis (MAP) caused by biallelic mutations in the *MUTYH* gene and NTHL1-associated polyposis caused by biallelic mutations in the *NTHL1* gene [4–7]. A timely identification of individuals at a high risk to develop CRC allows pre-symptomatic screening and genetic counseling, which may lead to reductions in both morbidity and mortality [8, 9].

In Western countries, CRC patients that are suspected of having a genetic predisposition for CRC are usually referred for genetic counseling to a clinical geneticist, who may advice for genetic testing. Until recently this testing was performed on a gene-by-gene basis, starting with the most likely candidate gene. With the advent of next-generation sequencing (NGS) technologies, however, it has become realistic to test a large panel of genes in a single assay, which is less laborious, less costly and less time-consuming [10]. Such an approach is also within reach now for centers and hospitals in which testing for a genetic cancer risk is not performed on a routine basis, as is the case in China.

Here, we describe a targeted next-generation sequencing panel of molecular inversion probes (MIPs) to identify high-penetrance CRC predisposing mutations in early-onset or familial CRC patients from China. We designed customized MIPs for the coding regions of seven high-penetrance CRC susceptibility genes, i.e., *APC*, *MLH1*, *MSH2*, *MSH6*, *PMS2*, *MUTYH* and *NTHL1* (*POLE* and *POLD1* exonuclease domains were independently tested by Sanger sequencing), and evaluated the performance of these MIPs in the detection of genetically predisposed patients diagnosed before the age of 35.

## RESULTS

### Patient cohort characteristics

**Figure 1:**
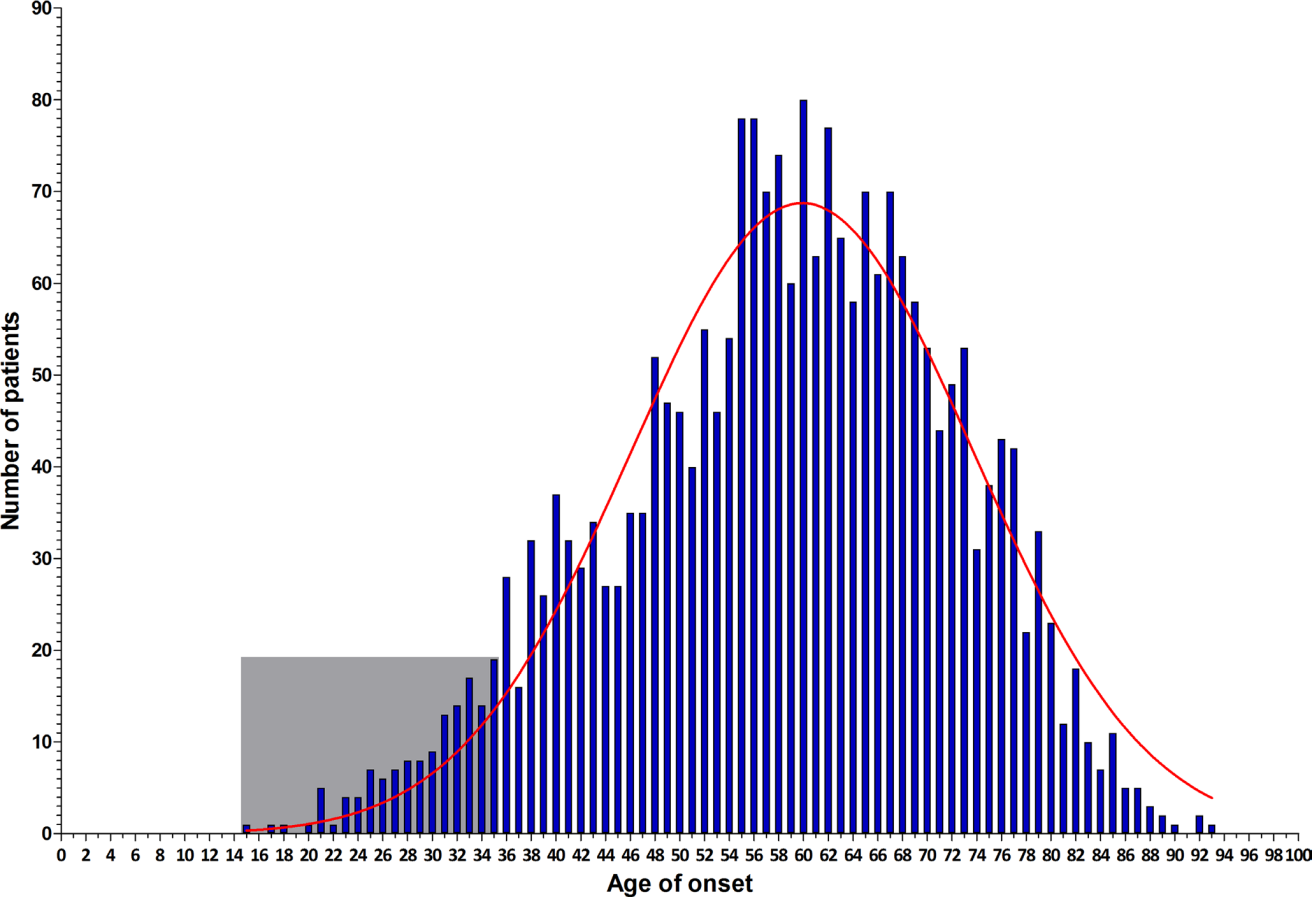
Age distribution histogram of the entire CRC patients series (*n* = 2,371) The frequency distribution per year-of-age in the entire CRC patient cohort is illustrated by blue bars, which show a normal distribution (red curve). The early-onset CRC patients enrolled for MIP-based sequencing are shown in the light gray square.

An unselected series of 2,371 CRC patients aged between 15 and 93 years (median age 59 years old) was collected (Figure 1). The majority of these patients was aged 48–73 years. From this series, patients diagnosed at or before the age of 35 years (early-onset) were selected for the current study, i.e., 140 cases of which 84 (60%) were male (Figure 1). The median age of onset was 31 years [range 15–35 years]. The demographic and clinical features of this cohort of 140 cases are listed in Table 1.

**Table 1:** Demographic and clinical characteristics of the study subjects

Characteristic	CRC diagnosed at ≤ 35 years	CRC diagnosed at > 35 years
Total patients	140 (5.9)	2,231 (94.1)
Age		
Median age of onset (range)	31 (15–35)	60 (36–93)
Mean age of onset	29.9	60.1
Gender		
Male (%)	84 (60.0)	1,312 (61.6)
Female (%)	56 (40.0)	819 (38.4)
Diagnosis		
Colon adenoma (%)^1^	8 (5.7)	33 (1.5)
Colon cancer (%)^2^	65 (46.4)	1,005 (45.1)
Rectal adenoma (%)^1^	2 (1.4)	14 (0.6)
Rectal cancer (%)^2^	65 (46.4)	1,179 (52.8)

^1^At least three adenomas detected; ^2^In the absence of (multiple) adenomas.

### Performance of the MIP sequencing panel

**Figure 2:**
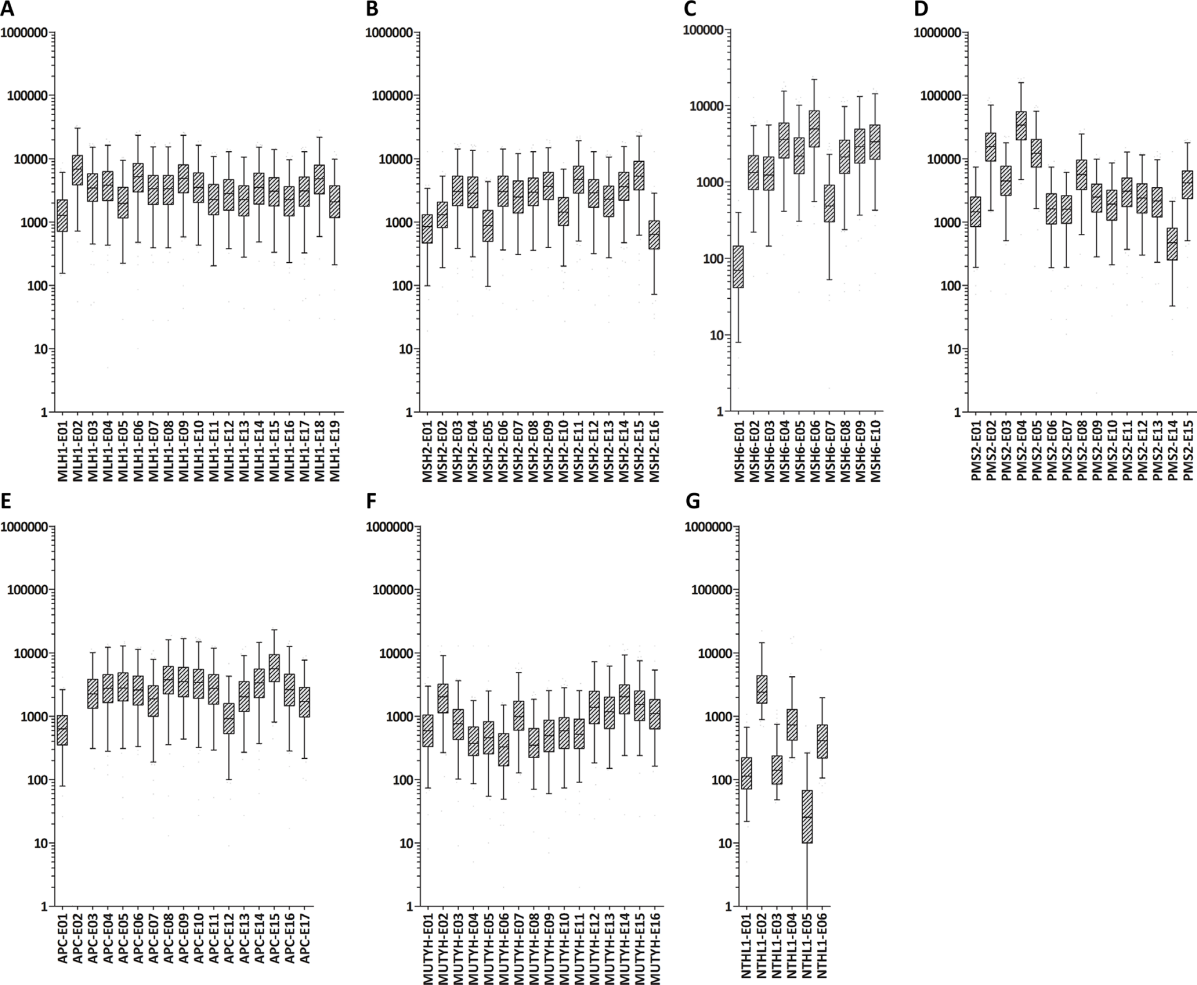
Mean read depth for each of the 99 exons targeted in the MIP panels Box plots of mean read depth of the targeted exons with boxes representing ±25% and whiskers depicting the 5–95% percentiles (Y-axis: fold coverage, x-axis: exons included for targeted sequencing). Mean read depth per exon of *MLH1* (**A**), *MSH2* (**B**), *MSH6* (**C**), *PMS2* (**D**), *APC* (**E**), *MUTYH* (**F**), and *NTHL1* (**G**).

The MIP sequencing panel was designed to cover all coding exons and intron-exon boundaries (+/−20 bp) of seven selected CRC predisposing genes with double tiling. Sequence capture and library preparation was performed for all 140 samples, using unique barcodes per sample. After sequencing, 99.0% of the targeted regions of interest (ROIs) were covered at least 10-fold. The mean read depth was 4,055x [range 50x-15,770x] for *APC*, *MLH1*, *MSH2*, *MSH6*, *PMS2* and *MUTYH* (‘six-gene’ panel) and 1,086x [range 63x-3,895x] for *NTHL1*. On average, 97.8% and 83.3% of the ROIs were covered >100x for the six-gene panel and for *NTHL1*, respectively (Figure 2). Three ROIs were not covered or had an average coverage of <100x, i.e., exon 2 of *APC* (no reads), exon 1 of *MSH6* (average coverage 92x) and exon 5 of *NTHL1* (average coverage 63x) (Figure 2).

### Identification of pathogenic germline variants in early-onset CRC cases

**Figure 3:**
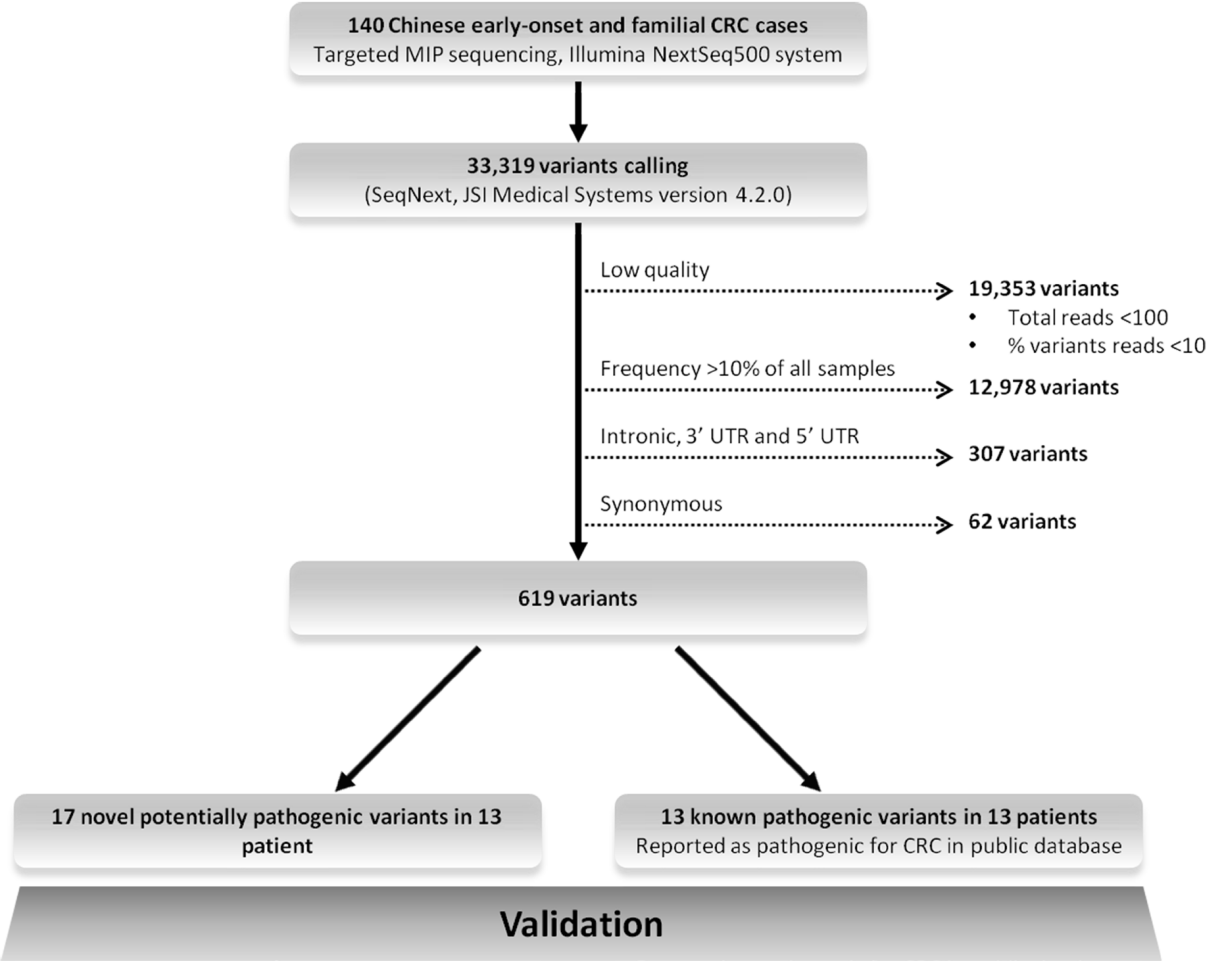
Schematic chart showing the stepwise strategy for the detection of pathogenic mutations Dashed arrows represent exclusion criteria. Germline variants known to be associated with hereditary CRC syndromes were initially selected and searched for evidence of pathogenicity in relevant databases, i.e., InSiGHT (http://www.insight-group.org/), LOVD (https://atlas.cmm.ki.se/LOVDv.2.0/), the Mismatch Repair Genes Variant Database (http://www.med.mun.ca/mmrvariants/) and Clinvar (http://www.ncbi.nlm.nih.gov/clinvar/). Next to the identification of known pathogenic variants, we searched for novel potential pathogenic rare variants (For details see Materials and Methods section).

**Figure 4:**
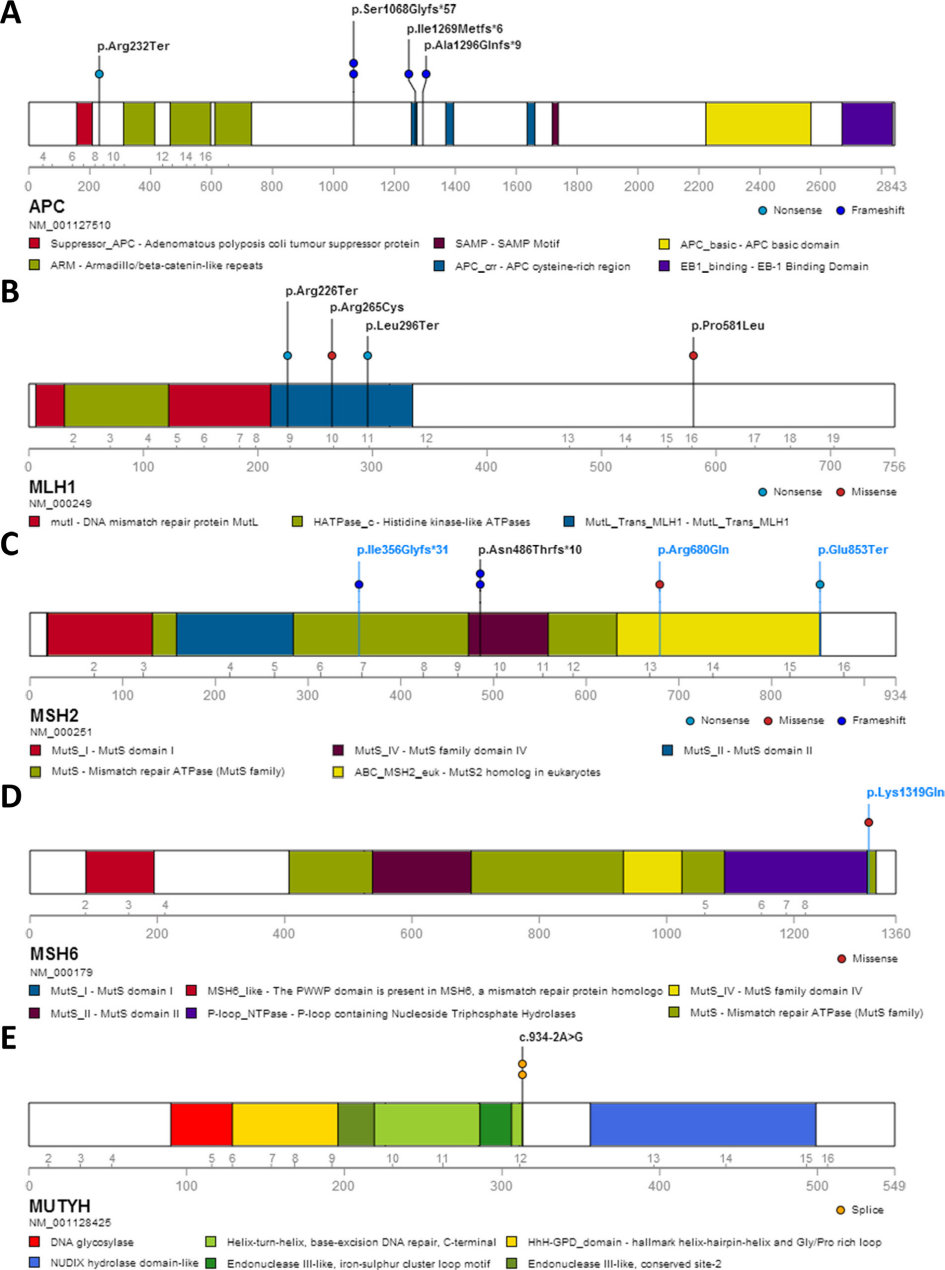
Protein alterations resulting from the mutations in known CRC predisposing genes identified in the studied cohort The alterations are shown for *APC* (**A**), *MLH1* (**B**), *MSH2* (**C**), *MSH6* (**D**) and *MUTYH* (**E**). Alterations shown in blue represent newly identified pathogenic mutations in this study; black entries denote previously reported pathogenic mutations in hereditary CRC syndromes. No loss of MSH6 protein expression was observed in the tumor of patient 14B-ON3654BD1, carrying the *MSH6* missense variant p.Lys1319Gln.

After a stringent filtering procedure (summarized in Figure 3), 30 candidate pathogenic variants were selected for further analysis. Of these 30 candidate variants, 17 were confirmed by Sanger sequencing, whereas the other 13 with either a low sequencing depth (< 300x) or a low percentage of variant reads (< 20%) appeared to be false-positives (Supplementary Table 1). Among the 17 validated pathogenic variants (Figure 4), 13 were previously reported in the LOVD or ClinVar databases as pathogenic mutations underlying the respective hereditary CRC syndromes (Table 2). Five germline mutations were identified in the *APC* gene, including one nonsense mutation (c.694C>T, p.Arg232Ter) and four frameshift mutations (c.3202_3205delTCAA, p.Ser1068Glyfs*57 (*n* = 2), c.3807_3808delAT, p.Ile1269Metfs*6 and c.3885delA, p.Ala1296Glnfs*9). Two probands carried a monoallelic splice site mutation (c.934-2A>G, p.Glu313Serfs*8) in the *MUTYH* gene, which was previously reported as potentially pathogenic in Japanese and Korean CRC patients [11, 12]. In addition, we found two known nonsense (c.676C>T, p.Arg226Ter and c.887T>G, p.Leu296Ter) and two pathogenic missense (c.793C>T, p.Arg265Cys and c.1742C>T, p.Pro581Leu) mutations in the *MLH1* gene, and a frameshift mutation (c.1457_1460delATGA, p.Asn486Thrfs*10) in the *MSH2* gene in two probands (Table 2). The remaining four variants, detected in the *MSH2* (*n* = 3) and *MSH6* (*n* = 1) genes, were not found to be present in the dbSNP, ESP and ExAC databases and were, therefore, subjected to further analysis (see below). No pathogenic mutations were identified in the *NTHL1* gene, and none of the genes was affected by germline copy number alterations using the CoNVaDING (Copy Number Variation Detection In Next-generation sequencing Gene panels) tool [13].

**Table 2:** Known germline pathogenic variants in CRC predisposing genes for early-onset CRC

Gene	Proband (gender)	Age	Diagnosis	Family history	cDNA change	Protein change	%Var. Reads	Var. Reads	Total Reads	LOVD^b^	MMR^c^	ClinVar^d^	InSiGHT^e^
*APC* (NM_001127510.2, NP_001120982.1)	12B-ON1914BD1 (M)	32	Tubular adenoma of colon (multiple)	Father CRC and death; uncle CRC and death, grandmother CRC and death	c.694C>T	p.Arg232Ter	38	1808	4758	62 +/+	NR	Path.	NA
	13B-ON3307BD1 (F)	25	Rectal polyps and rectal cancer	NA	c.3202_3205delTCAA	p.Ser1068Glyfs*57	25	614	2456	65 +/+	NR	Path.	NA
	14B-ON3493BD1 (F)	33	Rectal cancer	NA	c.3202_3205delTCAA	p.Ser1068Glyfs*57	29	735	2534	65 +/+	NR	Path.	NA
	13B-ON2394BD1 (F)	26	Colon polpys (multiple)	Mother FAP	c.3807_3808delAT	p.Ile1269Metfs*6	50	820	1640	2 +/+	NR	Path.	NA
	12B-ON1849BD1 (F)	23	Tubular adenoma of colon (multiple)	Mother FAP at 45 yr and death; old brother FAP at 22 yr and death; young brother FAP at 22 yr	c.3885delA	p.Ala1296Glnfs*9	32	529	1653	1 +/+	NR	NR	NA
*MLH1* (NM_000249.2, NP_000240.1)	H38 (M)	34	Rectal cancer	NA	c.676C>T	p.Arg226Ter	33	3255	9864	27 ?/?, 18 +/?, 2 +/+	NR	Path.	NA
	B838 (F)	29	Rectal cancer	NA	c.793C>T	p.Arg265Cys	39	823	2110	11 –/?, 1 –?/?, 28 ?/?, 4, +?/?, 19 +/?, 1 +/+	8 Inc., 14 NonPath., 14 Path.	Path.	Class 5
	B1366 (M)	32	Sigmoid colon cancer	NA	c.887T>G	p.Leu296Ter	39	260	667	2 ?/?	NR	Path.	Class 5
	14B-ON3619BD1 (M)	31	Sigmoid colon cancer	NA	c.1742C>T	p.Pro581Leu	47	2681	5704	1 –/?, 8 ?/?, 3 +/?	1 Inc., 1 NonPath., 3 Path.	Path.	Class 2
*MSH2* (NM_000251.2, NP_000242.1)	12B-ON1922BD1 (M)	34	Colon cancer	NA	c.1457_1460delATGA	p.Asn486Thrfs*10	27	1734	6422	15 ?/?, 1 +/+	NR	Path.	Class 5
	12B-ON2092BD1 (M)	23	Colon cancer	Mother rectal cancer and uncle colon cancer	c.1457_1460delATGA	p.Asn486Thrfs*10	33	159	482	15 ?/?, 1 +/+	NR	Path.	Class 5
*MUTYH* (NM_001128425.1, NP_001121897.1)	13B-ON3015BD1 (M)	21	Rectal cancer and anal cancer	NA	c.934-2A>G	p.(Glu313Serfs*8)^a^	43	79	184	4 ?/+?, 3 +/+?	NR	NR	NA
	13B-ON2469BD1 (M)	31	Sigmoid colon cancer	NA	c.934-2A>G	p.(Glu313Serfs*8)^a^	33	72	218	4 ?/+?, 3 +/+?	NR	NR	NA

### Characterization of novel germline variants in early-onset CRC cases

Two novel truncating mutations and one novel missense mutation in the *MSH2* gene (c.1062_1066delCAGAA, p.Ile356Glyfs*31, c.2557G>T, p.Glu853Ter and c.2039G>A, p.Arg680Gln) were identified in patients 13B-ON2505BD1 (male, rectal cancer at the age of 32 years), 13B-ON2469BD1 (male, sigmoid colon cancer at the age of 31 years) and B1287 (female, rectal cancer at the age of 30 years), respectively. One novel missense mutation in the *MSH6* gene (c.3955A>C, p.Lys1319Gln) was identified in patient 14B-ON3654BD1 (female, rectal cancer at the age of 34 years). Both missense variants were predicted to be pathogenic by the SIFT, Polyphen2 and CADD algorithms (Table 3).

**Table 3:** Novel germline variants in known CRC predisposing genes

Gene	Proband (gender)	Age	Diagnosis	Family history	cDNA change	Protein change	%Var. Reads	Var. Reads	Total Reads	phyloP	SIFT	PolyPhen2	CADDa
*MSH2* (NM_000251.2, NP_000242.1)	13B-ON2505BD1 (M)	32	Rectal cancer	NA	c.1062_1066delCAGAA	p.Ile356Glyfs*31	18	599	3328				35
	13B-ON2469BD1 (M)	31	Sigmoid colon cancer	NA	c.2557G>T	p.Glu853Ter	16	369	2306				40
	B1287 (F)	30	Rectal cancer	NA	c.2039G>A	p.Arg680Gln	15	309	2060	6.34	Del	PRD	35
*MSH6* (NM_000179.2, NP_000170.1)	14B-ON3654BD1 (F)	34	Rectal cancer	NA	c.3955A>C	p.Lys1319Gln	39	260	667	5.21	Del	POD	23.2

**Figure 5:**
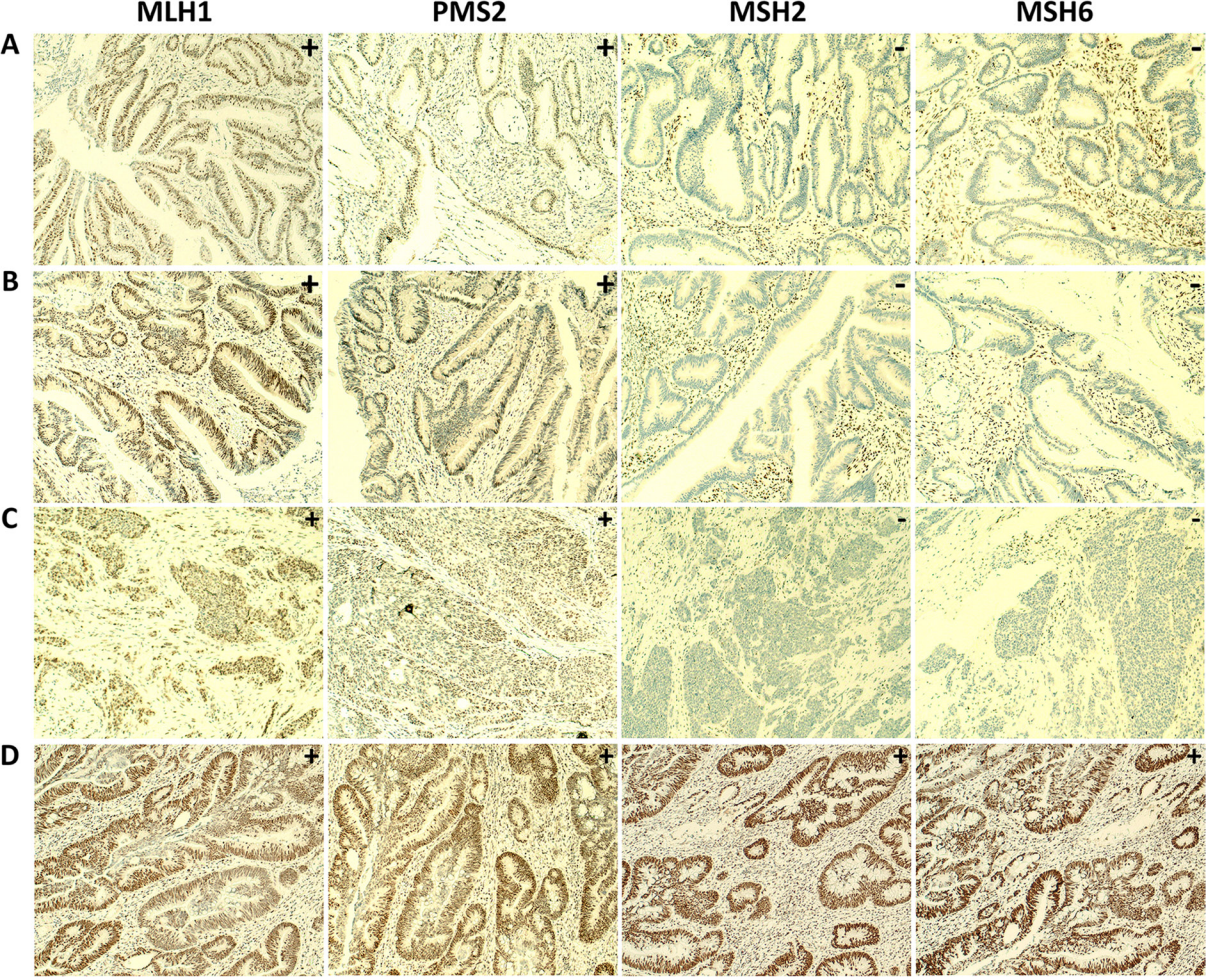
IHC staining of MMR proteins in tumor tissue of four patients carrying rare and novel germline mutations Loss of nuclear expression of the MSH2 and MSH6 proteins and normal expression of the MLH1 and PMS2 proteins in tumor tissues were observed in (**A**) patient 13B-ON2505BD1, with the *MSH2* p.Ile356Glyfs*31 frameshift mutation, (**B**) patient B1287, with the *MSH2* missense mutation p.Arg680Gln and (**C**) patient 13B-ON2469BD1, with the *MSH2* nonsense mutation p.Glu853Ter. Normal staining of the MLH1, PMS2, MSH2 and MSH6 proteins was observed in (**D**) tumor tissue of patient 14B-ON3654BD1, with the *MSH6* missense mutation p.Lys1319Gln. (100x magnification).

To assess whether the four novel MMR gene variants were indeed pathogenic, we performed immunohistochemistry (IHC) on the respective tumor tissues using antibodies directed against the MLH1, PMS2, MSH2 and MSH6 proteins. This analysis is based on the notion that a somatic second-hit mutation in the wild-type allele will result in loss of MLH1 and PMS2 expression in *MLH1*-mutated cases, loss of both MSH2 and MSH6 expression in *MSH2*-mutated cases and loss of MSH6 expression in *MSH6*-mutated cases [14, 15]. Formalin fixed paraffin embedded (FFPE) tissue blocks of seven cases were available for verification by IHC, including the four cases with novel mutations in the *MSH2* or *MSH6* genes. In the tumors derived from patients B838 and B1366, immunostaining was performed only for the MLH1 and MSH2 proteins. The remaining five patients’ tumor tissues were stained for all four MMR proteins (MLH1, MSH2, MSH6 and PMS2). Patients B838, B1366 and 14B-ON3619BD1, who carried known pathogenic variants in the *MLH1* gene, concordantly showed absence of MLH1 nuclear staining in their tumors. The tumor tissue from patient 14B-ON3619BD1 also showed a negative IHC staining for PMS2 (Table 4, Supplementary Figure 1). The three cases with novel mutations in the *MSH2* gene (13B-ON2505BD1, B1287 and 13B-ON2469BD1) all showed loss of expression of both the MSH2 and MSH6 proteins in their tumor tissues, which strongly suggests that these variants are indeed pathogenic. In patient 14B-ON3654BD1, carrying a novel missense variant in the *MSH6* gene, a normal expression of all MMR proteins was observed in the tumor tissue, indicating that this mutation may not be pathogenic (Table 4, Figure 5).

**Table 4:** Immunohistochemical expression of DNA MMR proteins in tumor tissue of patients carrying MMR gene mutations

Gene	Proband	Gene	Protein change	Tumor expression of MMR proteins			
				MLH1	PMS2	MSH2	MSH6
*Known germline pathogenic mutations*	H38	*MLH1*	p.Arg226Ter	N/A	N/A	N/A	N/A
	B838	*MLH1*	p.Arg265Cys	–	N/A	+	N/A
	B1366	*MLH1*	p.Leu296Ter	–	N/A	+	N/A
	14B-ON3619BD1	*MLH1*	p.Pro581Leu	–	–	+	+
	12B-ON1922BD1	*MSH2*	p.Asn486Thrfs*10	N/A	N/A	N/A	N/A
	12B-ON2092BD1	*MSH2*	p.Asn486Thrfs*10	N/A	N/A	N/A	N/A
*Rare and novel germline pathogenic mutations*	13B-ON2505BD1	*MSH2*	p.Ile356Glyfs*31	+	+	–	–
	B1287	*MSH2*	p.Arg680Gln	+	+	–	–
	13B-ON2469BD1	*MSH2*	p.Glu853Ter	+	+	–	–
	14B-ON3654BD1	*MSH6*	p.Lys1319Gln	+	+	+	+

*N/A*: Not available.

### Contribution of known and novel germline pathogenic mutations

**Figure 6:**
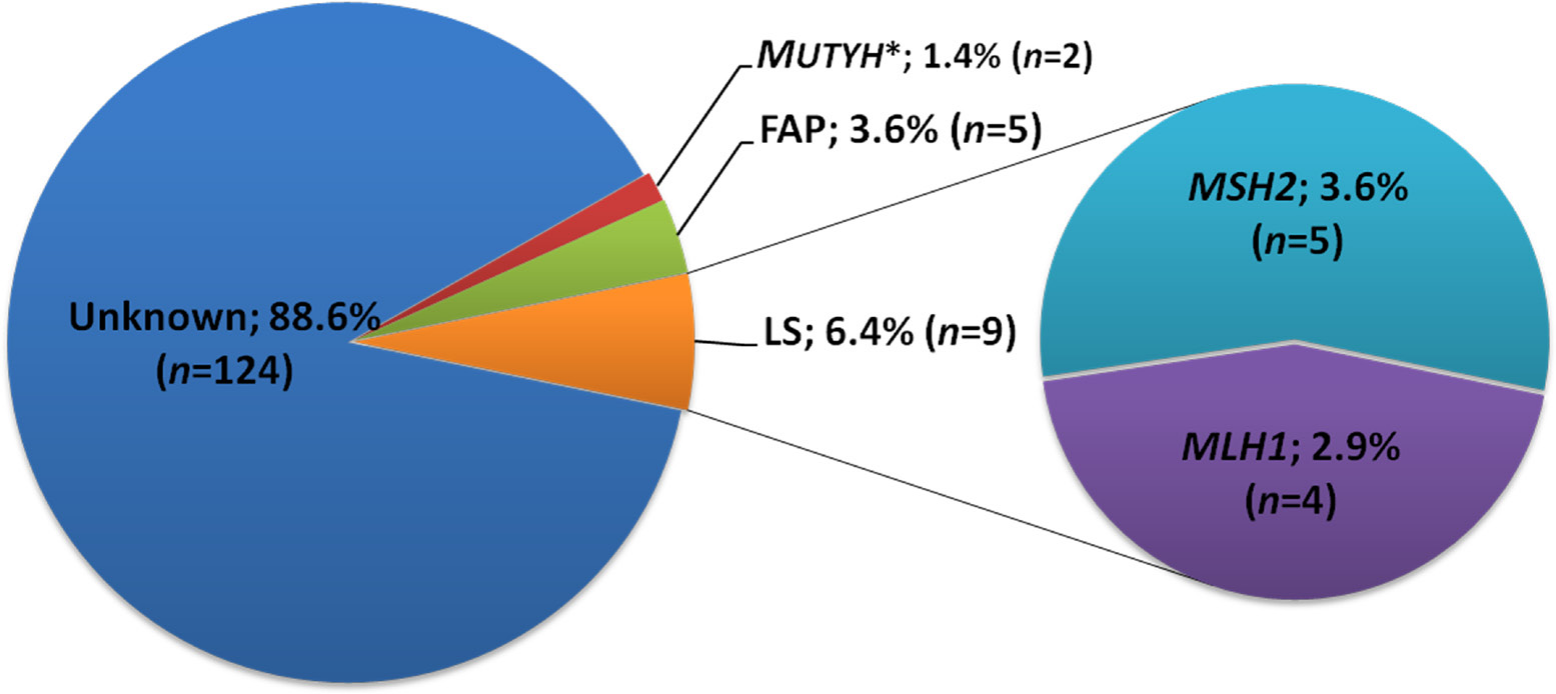
Contribution of known and novel germline pathogenic mutations to early-onset colorectal cancer in Chinese patients *In both *MUTYH* mutated patients (13B-ON3015BD1 and 13B-ON2469BD1, unrelated) only monoallelic mutations (c.934-2A>G; p.Glu313SerfsX8) were detected; FAP: familial adenomatous polyposis; LS: lynch syndrome.

Taken together, we identified pathogenic or likely pathogenic germline mutations in 16 of the 140 patients tested, and firmly established a diagnosis in 14 patients (10%) (Figure 6). The most frequently diagnosed syndrome was Lynch Syndrome (9 cases; 6.4%). Four patients (2.9%) carried a mutation in the *MLH1* gene and five patients (3.6%) carried a mutation in the *MSH2* gene. FAP, caused by mutations in *APC*, was diagnosed in 3.6% (5 cases) of the patients. Two patients carried a monoallelic mutation in the *MUTYH* gene, but mutations in the remaining allele, which would be indicative for the diagnosis of MAP, were not detected.

## DISCUSSION

A targeted next-generation sequencing panel using molecular inversion probes (MIPs) was developed to identify high-penetrance mutations in the CRC predisposing genes *APC*, *MLH1*, *MSH2*, *MSH6*, *PMS2*, *MUTYH* and *NTHL1*. We applied this panel to a cohort of 140 early-onset Chinese CRC patients (diagnosed at or below the age of 35 years). Using this approach, we identified known pathogenic mutations in 13 cases, and in three additional cases novel, likely pathogenic, mutations in the *MSH2* gene were found with confirmed loss of MSH2 protein expression in the tumors. Two of the 13 patients were found to carry a known pathogenic monoallelic *MUTYH* mutation, of which the clinical relevance remains inconclusive at this point. Therefore, a firm diagnosis could be made in 14 cases (10%).

The diagnosis hereditary CRC mainly relies on clinico-pathological features, family history and genetic data. Although international criteria for hereditary CRC have been defined, such as the Amsterdam criteria I and II, the Bethesda guidelines and the Japanese standards, they are not suitable for small pedigrees, which are common in China. Furthermore, the characteristics of hereditary CRC cases in the Chinese population may differ from those in Western countries [16]. As of yet, genetic testing for hereditary CRC is not performed on a routine basis in China. The recent development and implementation of next-generation sequencing technologies makes rapid and cost-effective genome sequencing feasible. Particularly, targeted sequencing approaches are highly suitable for implementation in routine diagnostic testing of patients with a suspected (colorectal) cancer syndrome. Here, we used MIP-based sequencing, a recently developed approach that has successfully been applied to targeted DNA sequencing of clinical samples [17, 18]. MIPs provide a high target sensitivity (99%) and specificity (98%) at low costs and minimal DNA requirements, and can be easily multiplexed to target regions of multiple genes in a single reaction [18]. Implementation of this methodology in routine diagnostics requires further adjustments to guarantee minimal costs and optimal turnaround times, which are highly dependent on sample supply, available infrastructure, and local personnel costs [19, 20]. For a MIP-based breast cancer susceptibility gene panel, a turnaround time of 4 days has been described [19]. Therefore, we consider MIP-based sequencing as a highly suitable replacement of Sanger sequencing for clinical genetic testing [17].

We found that with our newly designed MIP sequencing panel 99% of the target regions of interest (ROIs) were covered at least 10x. The majority (97%, 96/99) of the target ROIs was covered more than 100x (ranging from 186x to 15,770x), whereas exon 2 of *APC*, exon 5 of *NTHL1* and exons 1 of *MSH6* showed poor coverages. For *APC*, no pathogenic or likely pathogenic mutations in exon 2 associated with CRC or FAP have been reported in the LOVD [21]. Exon 5 of *NTHL1* showed a mean coverage of 63x and exon 1 of *MSH6* showed a mean coverage of 92x. Further probe rebalancing and supplementation may improve the capture uniformity from probe to probe and the coverage of the poorly captured regions. For the candidate pathogenic variants, Sanger sequencing-based validation revealed that 17 variants with a good coverage (> 600x) and a high mutant allele frequency (≥ 25%) could readily be confirmed, while the other 13 discrepant variants with a relatively low sequencing depth (< 300x) or a low variant allele frequency (< 20%) could not be confirmed (Supplementary Table 1), suggesting that further optimization of the thresholds for read depth and variant allele frequency will lower the number of false positives. The use of a higher thresholds for variant calling may decrease the chance to detect patients with (rarely occurring) germline mutations that are present in a low-mosaic state [22]. To overcome this limitation, we recently applied single-molecule molecular inversion probes (smMIPs) to FFPE tissue-derived DNA, which performs molecular tagging of DNA molecules, and gives reliable results [20].

Of note, among the 17 confirmed mutations identified, 13 (76.5%) were previously reported to be associated with hereditary CRC syndromes in public databases such as InSiGHT, LOVD and the Mismatch Repair Genes Variant database. Three novel mutations in the *MSH2* gene were considered to be likely pathogenic, whereas one novel missense variant in the *MSH6* gene may not be pathogenic based on our IHC staining results. Together, these pathogenic and likely pathogenic germline mutations explain 10% of the early-onset CRC patients included in our cohort. Some of the remaining 90% of the patients in our cohort may carry germline mutations in known genes that are not included in our panel yet, such as *SMAD4/BMPR1A*, *POLE* or *POLD1*, or in genes that still await discovery. However, it is likely that the majority of these cases does not carry a mutation in a high-penetrant gene. Lower penetrance genetic factors and/or environmental factors may have caused the early onset of colorectal cancer in these cases, as has recently been proposed [23].

With five pathogenic germline mutations in five patients, *APC* is the most frequently mutated CRC predisposition gene in our cohort. Indeed, from four of these patients a polyposis phenotype (> 100 colonic polyps) had indeed been recorded. With mutations detected in 6 MMR genes, Lynch Syndrome is the most prevalent CRC syndrome encountered in our cohort, which is in agreement with a previous study using whole exome sequencing of Chinese early-onset and familial CRC patients [24] and with the studies of Tanskanen et al. [25] and Chubb et al. [23]. Others have reported various frequencies of germline MMR gene mutations in selected CRC cohorts with a broad range in estimates of their contributions to CRC development [26]. *MSH2* was found to be the most frequently mutated MMR gene in our cohort, which is consistent with other studies in southern Chinese CRC patients reported by Liu et al. [27], Yang et al. [28] and Jin et al. [29]. However, this frequency differs from other studies reporting that the *MLH1* gene was more frequently affected than the *MSH2* gene [24, 30–32]. More studies are, however, required to exactly determine the *MLH1* and *MSH2* mutation rates in different regions and ethnicities in China.

We identified in two unrelated patients a monoallelic *MUTYH* splice site variant (c.934-2A>G, p. Glu313SerfsX8), which has been reported to be probably pathogenic in Japanese and Korean CRC patients [11, 12]. Although no mutations were identified on the second *MUTYH* allele in these two individuals, it is still possible that pathogenic germline mutations are present on this allele outside the detection range of our MIP sequencing panel (i.e., introns or promoter). Sequencing of tumor-derived DNA of these patients may additionally reveal whether the somatic mutations present are biased towards G>A transversions, which is a typical feature of MAP-associated tumors [33].

In summary, we developed a MIP-based next-generation sequencing panel that targets the coding exons and flanking exon-intron boundaries (+/−20 bp) of seven high-penetrant CRC predisposing genes. Our data indicate that targeted MIP-based sequencing is a reliable approach for the identification of Chinese early-onset CRC patients with a Mendelian syndrome. This approach is cost- and time-efficient compared to Sanger sequencing [18]. For the specific purpose of molecular diagnostics, this strategy shows advantages over phenotype-based gene-specific testing. Since the international standards on hereditary CRC are not applicable to small families as commonly seen in China, such a sequencing-based strategy may be particularly beneficial for the Chinese population.

## MATERIALS AND METHODS

### Study subjects

Whole blood from 2,391 patients with CRC was collected between 2007 and 2014 by the Sixth Affiliated Hospital, Sun Yat-sen University, Guangzhou, China. Probands with an age at diagnosis of ≤ 35 years, with or without a family history for CRC, were selected. For the current study 140 early-onsets CRC cases were included of whom DNA was available for testing. All included patients provided informed consent. This study was reviewed and approved by the Institutional Review Board of the Sixth Affiliated Hospital, Sun Yat-sen University, Guangzhou, China.

### Genomic DNA isolation

Genomic DNA was extracted from peripheral blood cells using a Gentra Puregene Blood Kit (QIAGEN, Hilden, Germany) according to the protocol provided by the manufacturer. The DNA was quantified using a NanoDrop 2000 UV-Vis Spectrophotometer (Thermo Scientific) and the DNA concentration was normalized to 25 ng/μl for MIP-based sequencing.

### Molecular inversion probe (MIP) design, capture and sequencing

For six genes included in the targeted sequencing effort in this study, i.e., *APC*, *MLH1*, *MSH2*, *MSH6*, *PMS2* and *MUTYH*, 626 MIPs were designed. In addition, 23 MIPs targeting the last exon of *EPCAM*, a gene located ∼25 kb upstream of *MSH2*, were included to allow detection of deletions of the 3′ end of *EPCAM*, which leads to *in cis* inactivation of the *MSH2* promoter [34] (Supplementary Figure 2). For the *NTHL1* gene we generated another multiplex panel of 23 MIPs (Table 5). These MIPs were designed as described previously [35], targeting the coding exons and flanking exon-intron boundaries (+/−20 bp) of the respective genes, in total covering 22,834 of the 23,062 (99.01%) coding base pairs. Except for *NTHL1*, all targeted regions had a double tiling. Targeted capture with MIPs was performed as previously reported [18, 36], with some slight modifications [7]. In brief, a total of 100 ng of genomic DNA was used to capture the target regions in a mixture of 25 μl containing 4.38E-05 picomoles 5′-phosphorylated MIPs in Ampligase DNA Ligase Buffer (Epicentre, Madison, WI, USA), 0. 32 μM dNTPs (NEB, Ipswich, MA, USA), 3.2 U Hemo Klentaq (NEB, Ipswich, MA, USA), and 1 U Ampligase 100 U/ul (Epicentre, Madison, WI, USA). After denaturation (95°C) for 10 minutes and incubation (60°C) for 22 hours, linear probes and the remaining genomic DNA were removed by exonuclease treatment. Next, the captured material was amplified by PCR using barcoded reverse primers. The resulting PCR products were pooled and combined (140 samples) into a library. The resulting libraries (from the two multiplex panels) were sequenced using an Illumina NextSeq500 system, with 2 × 150-bp paired-end reads.

**Table 5:** Summary of 7 high-penetrance CRC genes in the MIP panel

Gene	Gene Acession Number	# of Exons	# of Coding Bases	# of Targeted Coding Bases	# of MIPs	Associtated Syndromes^c^	Associated Cancers^d^ (Lifetime risk (%) or fold-increase in risk)	NCCN Guidelines [48]	References
APC	NM_001127510.2	17	8725	8618	201	Familial Adenomatous Polyposis (FAP)/Attenuated FAP (AFAP)	Colorectal (70% for AFAP, 100% for FAP), Duodenum/periampullary (4–12%), Thyroid (1–2%), Hepatoblastoma (1–2), Pancreas (2%), Medulloblastoma (< 1%), Gastric (< 1%)	Y	[48–51]
MLH1	NM_000249.2	19	2271	2218	86	Lynch Syndrome	Colorectal (15–82%), Endometrial (15–60%), Gastric (11–19%), Ovarian (9–12%), Small intestine, Hepatobiliary, Upper urinary tract, CNS, Sebaceous gland	Y	[52–57]
MSH2^a^	NM_000251.2	16	2805	2750	112				
MSH6	NM_000179.2	10	4083	4070	101				
PMS2	NM_000535.5	15	2589	2589	84				
MUTYH	NM_001128425.1	16	1650	1650	65	MUTYH-associated Polyposis (MAP)	Colorectal (80%), Breast cancer (females), Duodenum (4%), Gastric, Endometrial	Y	[58–61]
NTHL1^b^	NM_002528.5	6	939	939	23	NTHL1-associated Polyposis	Colorectal and Endometrial	N	[7]
Total		99	23062	22834	672				

### Data analysis and variant calling

Barcode-specific FASTQ files were mapped and annotated for *APC* (NM_001127510.2), *MLH1* (NM_000249.2), *MSH2* (NM_000251.2), *MSH6* (NM_000179.2), *PMS2* (NM_000535.5), *MUTYH* (NM_001128425.1) and *NTHL1* (NM_002528.5), and variants were called using SeqNext (JSI Medical Systems; version 4.2.0). Subsequently, we selected all variants not found in our in-house database (5,036 in-house analyzed exomes, without suspected cancer predisposition, mostly from European ancestry), and with a MAF < 0.001 in dbSNPv142, Exome Variant Server, NHLBI GO Exome Sequencing Project (ESP), Seattle, WA (6,503 exomes, URL: http://evs.gs.washington.edu/EVS/) and Exome Aggregation Consortium (ExAC), Cambridge, MA (60,706 unrelated exomes, http://exac.broadinstitute.org). To exclude false-positive calls due to technical artifacts, the following variant calls were excluded: less than 100-fold absolute coverage, less than 10% variant reads and less than 30 variant reads. Furthermore, all unknown variants that were called in > 10% of the samples were considered as local normal variation and were also excluded.

### Delineation of pathogenic mutations

A stepwise strategy was used to systematically identify putative pathogenic mutations. We initially selected germline variants known to be associated with hereditary CRC syndromes and searched for evidence of pathogenicity in relevant databases, i.e., InSiGHT (http://www.insight-group.org/), LOVD (https://atlas.cmm.ki.se/LOVDv.2.0/), the Mismatch Repair Genes Variant Database (http://www.mmruv.info/) and ClinVar (http://www.ncbi.nlm.nih.gov/clinvar/). Next to the identification of known pathogenic variants, we searched for novel potential pathogenic rare variants using the integrated mutation prediction software Alamut Visual version 2.5 (Interactive Biosoftware, Rouen, France). For the selection of these variants, at least one of the following criteria should be met: (i) variants that result in truncation of the protein, including nonsense and frameshift variants, and variants predicted to cause splice site defects; (ii) non-synonymous missense variants at highly conserved nucleotide positions (phyloP ≥ 3.0) [37], which score “deleterious” by both SIFT [38] and PolyPhen2 [39]; (iii) variants with CADD scores > 15 as scored by Combined Annotation-Dependent Depletion (CADD) [40], which is a method for objectively integrating diverse annotations into a single measure (C score) for each variant.

### Variant validation by sanger sequencing

The identified potentially pathogenic germline variants were validated by Sanger sequencing after PCR amplification. The PCR primers were designed using the Primer3 software package [41] (primer sequences available upon request). The PCR reactions were performed using a Dual 96-Well GeneAmp PCR System 9700 (Applied Biosystems) using standard protocols. Mutation analyses were performed using the Vector NTI software package (Invitrogen, Paisley, UK).

### Immunohistochemical detection of MMR proteins

Expression of the MMR proteins MLH1, MSH2, MSH6 and PMS2 was assessed by immunohistochemistry (IHC) on 4-μm sections of formalin fixed paraffin embedded (FFPE) tissue samples containing tumor tissue and adjacent normal mucosa. IHC staining was performed using a BenchMark XT automated tissue staining system (Ventana Medical Systems, Inc., Tucson, AZ, USA), according to validated protocols provided by the manufacturer. The antibodies used were a mouse anti-MLH1 monoclonal antibody (clone ES05; dilution 1:100; ZSGB-BIO), a rabbit anti-MSH2 monoclonal antibody (clone RED2; dilution 1:150; ZSGB-BIO), a rabbit anti-MSH6 monoclonal antibody (clone EP49; dilution 1:200; ZSGB-BIO), and a rabbit anti-PMS2 monoclonal antibody (clone EP51; dilution 1:40; ZSGB-BIO). The tissue sections were counterstained with hematoxylin. Nuclear immunoreactions in lymphocytes, normal colonic mucosa cells or stromal cells within the tissue sections served as internal positive controls. As an external positive control, normal colon tissue was used. The staining of the sections was independently evaluated by two experienced GI pathologists (X.J.F, W.Y.T).

### Copy number variations (CNVs) analysis

Copy number variations (CNVs) were evaluated using the CoNVaDING (Copy Number Variation Detection In Next-generation sequencing Gene panels) tool for detecting single exon CNVs in targeted NGS data [13]. In brief, the pooled raw sequence data were first demultiplexed, extracting reads per sample. For each sample the sequence data were aligned to the human reference genome build 37, as released by the 1000 Genomes project [42], using BWA [43]. The resulting BAM files were utilized for CNV calling and genotyping, and post-processing was performed using CoNVaDING [13]. This algorithm firstly calculates the average depth of coverage for each target, and then selects the control samples showing the most similar coverage pattern from a set of possible control samples based on the match quality control (QC) metric, thereby limiting the sample-to-sample variation. In order to obtain a CNV call, the depth of coverage has to differ significantly from the average of two alternative normalizations, using either all (autosomal) targets or all targets belonging to the same gene. CNVs were called by a logical combination of the different ratio score that captures the relative coverage difference between samples and controls, and the distribution score based on a Z-score calculation, which indicates whether a difference is significant or not. QC metrics were calculated for the samples and the targets, making explicit which targets are suitable for analysis with high sensitivity and specificity. Using the target QC values to filter CNVs, high quality calls are separated from calls that are more likely to be false positives.

## SUPPLEMENTARY MATERIALS FIGURES


