# Supplementary material for: A molecular inversion probe-based next-generation sequencing panel to detect germline mutations in Chinese early-onset colorectal cancer patients
Source: Oncotarget. 2017 Feb 21;8(15):24533–47. doi: 10.18632/oncotarget.15593 (PMC5421868; doi:10.18632/oncotarget.15593)
Supplement: Supplementary file 1 [file oncotarget-08-24533-s001.pdf]

A molecular inversion probe-based next-generation sequencing panel to detect germline mutations in Chinese early-onset colorectal cancer patients

Supplementary Materials

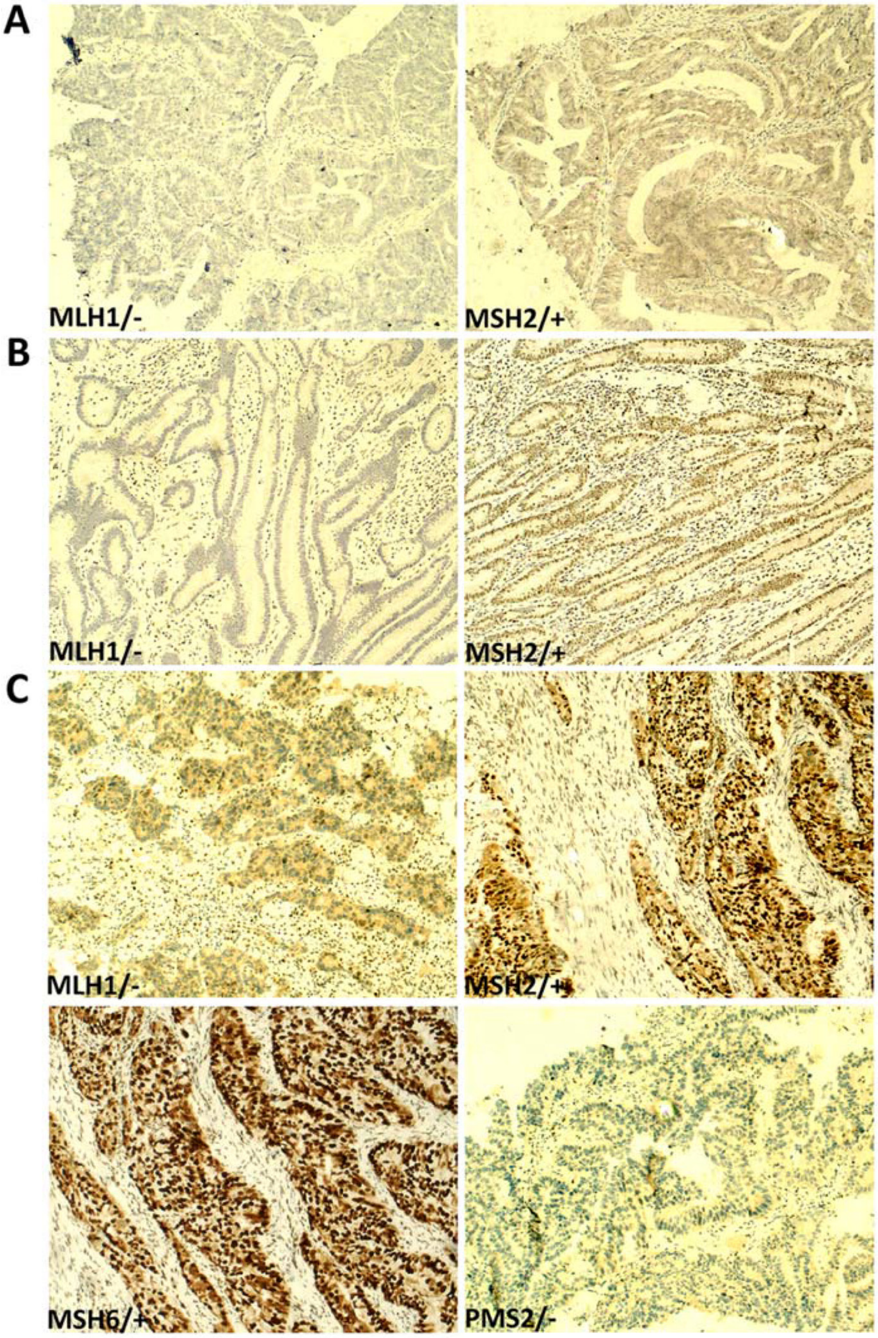

Supplementary Figure 1: IHC staining of DNA MMR proteins in tumor tissue of patients carrying known germline pathogenic mutations at 100x magnification. Loss of nuclear expression of the MLH1 protein and normal staining for the MSH2 protein in tumor tissue were observed in (**A**) patient B838, with missense mutation of MLH1 gene p.Arg265Cys, and (**B**) patient B1366, with nonsense mutation of MLH1 gene p.Leu296Ter; Loss of nuclear expression for MLH1 and PMS2 proteins and retained expression of MSH2 and MSH6 proteins in tumor tissue were observed in (**C**) patient 14B-ON3619BD1, with missense variant of MLH1 gene p.Pro581Leu.

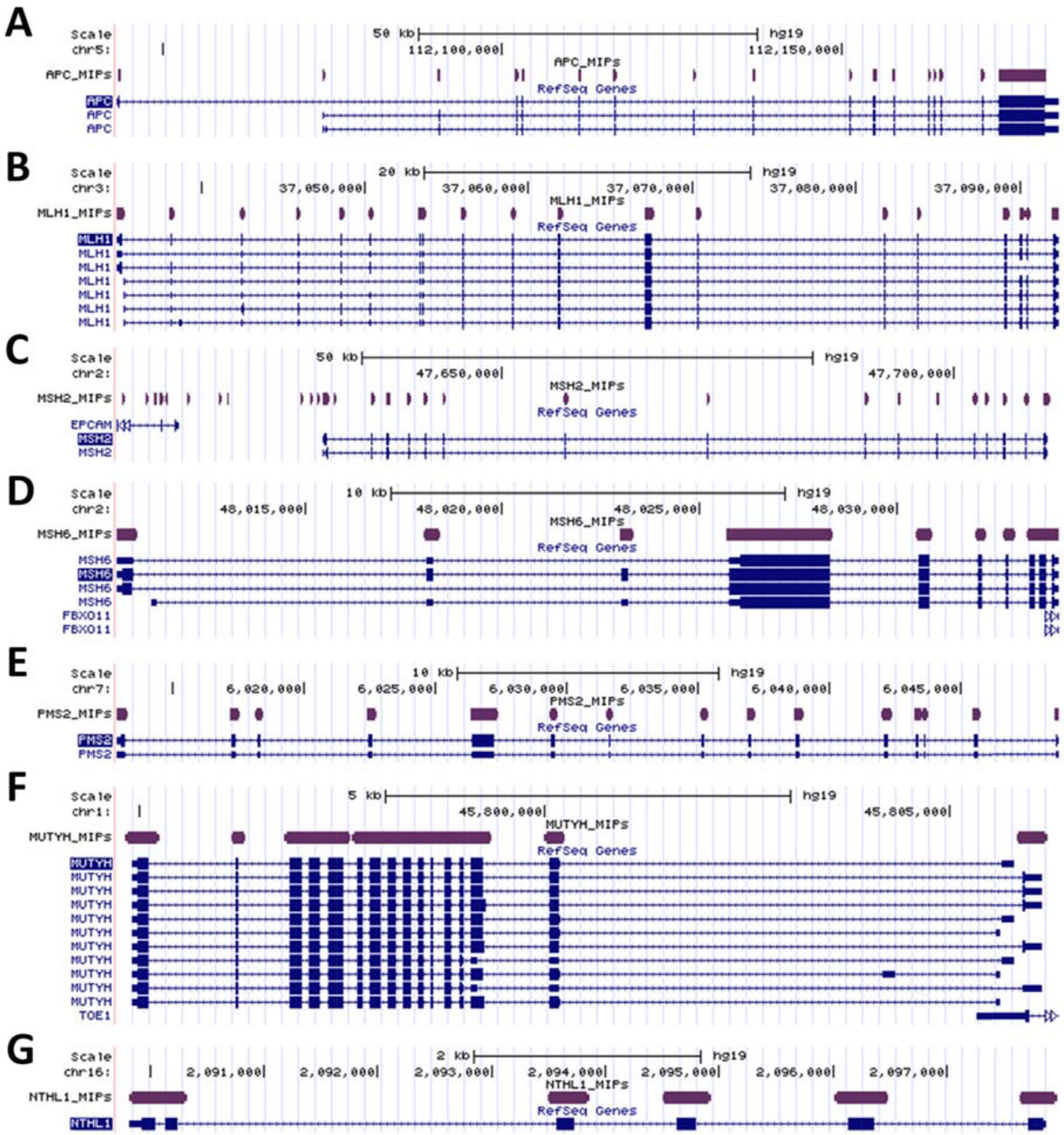

Supplementary Figure 2: The molecular inversion probes (MIPs) designed for the regions of interest are tracked and shown versus physical position on the UCSC Genome Browser in the Human February 2009 (GRCh37/hg19) genome release. Purple and blue ideograms indicated the region covered by the MIPs and the RefSeq genes respectively. (**A**) MIPs designed for *APC* gene. (**B**) MIPs designed for *MLH1* gene. (**C**) MIPs designed for *MSH2* gene. (**D**) MIPs designed for *MSH6* gene. (**E**) MIPs designed for *PMS2* gene. (**F**) MIPs designed for *MUTYH* gene. (**G**) MIPs designed for *NTHL1* gene.
